# Role of ICU-acquired weakness on extubation outcome among patients at high risk of reintubation

**DOI:** 10.1186/s13054-020-2807-9

**Published:** 2020-03-12

**Authors:** Arnaud W. Thille, Florence Boissier, Michel Muller, Albrice Levrat, Gaël Bourdin, Sylvène Rosselli, Jean-Pierre Frat, Rémi Coudroy, Emmanuel Vivier

**Affiliations:** 1 ALIVE Research group INSERM CIC 1402, University of Poitiers, Poitiers, France; 2 Médecine Intensive Réanimation, Centre Hospitalier Universitaire de Poitiers, Poitiers, France; 3 Réanimation Médicale, CHU de Poitiers, 2 rue la Milétrie, 86021 Poitiers Cedex, France; 4 Centre Hospitalier Annecy Genevoix, Réanimation Polyvalente, Metz-Tessy, France; 5 Centre Hospitalier Saint Joseph Saint Luc, Réanimation Polyvalente, Lyon, France

**Keywords:** Weaning, Extubation, Mechanical ventilation, ICU-acquired weakness, Intensive care unit

## Abstract

**Background:**

Whereas ICU-acquired weakness may delay extubation in mechanically ventilated patients, its influence on extubation failure is poorly known. This study aimed at assessing the role of ICU-acquired weakness on extubation failure and the relation between limb weakness and cough strength.

**Methods:**

A secondary analysis of two previous prospective studies including patients at high risk of reintubation after a planned extubation, i.e., age greater than 65 years, with underlying cardiac or respiratory disease, or intubated for more than 7 days prior to extubation. Patients intubated less than 24 h and those with a do-not-reintubate order were not included. Limb and cough strength were assessed by a physiotherapist just before extubation. ICU-acquired weakness was clinically diagnosed as limb weakness defined as Medical Research Council (MRC) score < 48 points and severe weakness as MRC sum-score < 36. Cough strength was assessed using a semi-quantitative 5-Likert scale. Extubation failure was defined as reintubation or death within the first 7 days following extubation.

**Results:**

Among 344 patients at high risk of reintubation, 16% experienced extubation failure (56/344). They had greater severity and lower MRC sum-score (41 ± 16 vs. 49 ± 13, *p* < 0.001) and were more likely to have ineffective cough than the others. The prevalence of ICU-acquired weakness at the time of extubation was 38% (130/244). The extubation failure rate was 12% (25/214) in patients with no limb weakness vs. 18% (12/65) and 29% (19/65) in those with moderate and severe limb weakness, respectively (*p* < 0.01). MRC sum-score and cough strength were weakly but significantly correlated (rho = 0.28, *p* < .001). After multivariate logistic regression analyses, the lower the MRC sum-score the greater the risk of reintubation; severe limb weakness was independently associated with extubation failure, even after adjustment on cough strength and severity at admission.

**Conclusion:**

ICU-acquired weakness was diagnosed in 38% in this population of patients at high risk at the time of extubation and was independently associated with extubation failure in the ICU.

## Background

The decision of extubation is a critical moment in the ICU because mortality is particularly high in case of extubation failure leading to reintubation [1, 2]. The overall rate of reintubation after planned extubation is around 10% but may exceed 20% in some subsets of patients [1, 2]. Identification of patients at high risk of reintubation deserves consideration in order to apply specific measures that may prevent reintubation. The most recent international clinical practice guidelines recommend the use of non-invasive ventilation immediately after extubation to prevent respiratory failure in patients at high risk of reintubation [3]. In patients older than 65 years or having any underlying cardiac or respiratory disease, a recent large randomized clinical trial showed that a combination of high-flow nasal oxygen alternating with non-invasive ventilation was the most efficient strategy to prevent reintubation [4, 5].

In addition to patient characteristics, other factors acquired during the ICU stay, such as ICU-acquired weakness or inability to clear secretions, may also be associated with increased risk of extubation failure. Several studies have shown that ineffective cough at the time of extubation is a strong predictor of reintubation [6–17]. Ineffective cough is mainly due to altered expiratory muscle function including at varying degrees of the diaphragm, accessory respiratory muscles, and abdominal muscles [18, 19]. ICU-acquired weakness combining polyneuropathy, myopathy, and muscle atrophy [20–22] is clinically diagnosed as limb paresis but may affect all respiratory muscles with an altered inspiratory and expiratory strength, as well pharyngeal muscles, and which can lead to overall respiratory muscle weakness [23, 24] and to swallowing disorders [25].

Whereas ICU-acquired weakness may lead to difficult weaning and delayed extubation in mechanically ventilated patients [23, 24, 26–28], few studies have assessed its role in extubation failure [29, 30]. To our knowledge, only one study has reported that patients with limb weakness have higher reintubation rates than others [30], but cough strength, a potential confounding factor, was not assessed, making the results uncertain.

Whether ICU-acquired weakness is a real risk factor for reintubation is unknown. Therefore, the primary objective of this study was to assess the influence of ICU-acquired weakness on extubation failure, in patients at high risk of reintubation in the ICU. Secondary objectives included assessment of the other factors associated with extubation failure, especially cough strength, and the relationship between limb weakness and cough strength.

## Methods

### Study design and participants

This is a secondary analysis of two previous prospective studies assessing extubation outcome in the ICU [15, 16]. The first study was a prospective monocenter cohort conducted in a teaching hospital between November 2010 and April 2012 [15]. The second was a prospective multicenter study conducted in three ICUs between March 2015 and October 2016 [16].

In the present study, only patients at high risk of reintubation were retained in the analysis, i.e., patients older than 65 years, with underlying chronic cardiac or respiratory disease, or intubated for more than 7 days prior to extubation [15, 31, 32]. Underlying chronic cardiac disease included left ventricular dysfunction whatever the cause defined by left ventricular ejection fraction ≤ 45%, history of cardiogenic pulmonary edema, documented ischemic heart disease, or permanent atrial fibrillation. Underlying chronic lung disease included chronic obstructive pulmonary disease, obesity hypoventilation syndrome, or restrictive pulmonary disease. Patients with limb weakness prior to ICU admission, patients intubated less than 24 h in the ICU, and those with a do-not-reintubate order upon extubation were not included. Post hoc analysis of data of the two original studies was approved by the two Ethics Committees (CPP Ile-de-France IX and CPP Sud-Est II).

### Weaning procedure

The decision to extubate was made by the attending physician in patients fulfilling all the weaning criteria after passing a spontaneous breathing trial [33]. Weaning criteria were considered as follows: respiratory rate ≤ 35 breaths per minute, adequate oxygenation defined as SpO_2_ > 90% with FiO_2_ ≤ 0.4 or PaO_2_/FiO_2_ > 150 mmHg with positive end-expiratory pressure (PEEP) ≤ 8 cmH_2_O, patient awake with a Richmond Agitation-Sedation Scale [34] between + 1 and − 2, no continuous sedation, and no need for vasopressors. The spontaneous breathing trial was performed either with T-piece or with low levels of pressure-support (PS 7 cmH_2_O) without positive end-expiratory pressure for 1 h [33].

Failure of the spontaneous breathing trial was defined according to the usual criteria of the international conference consensus on weaning from mechanical ventilation as the development during the trial of any of the following events [33]: respiratory rate > 35 breaths/min, increased accessory muscle activity, SpO_2_ persistently below 90% (on FiO_2_ 0.4 or at least 6 L/min of oxygen), heart rate persistently above 140 beats/min, systolic blood pressure below 90 or above 180 mmHg, or appearance of cyanosis or mottling, depressed mental status, or agitation.

### Clinical assessment of ICU-acquired weakness

Limb muscle and cough strength, as well as the amount of secretions, were assessed by a physiotherapist blind to the patient’s medical condition just before extubation. The inability to manage secretions, potentially leading to postponed extubation, was considered in patients with both ineffective cough and abundant secretions. Limb muscle strength was assessed using the Medical Research Council (MRC) score for the three muscle groups of each limb, with an overall score ranging from 0 (total paralysis) to 60 (normal muscle strength); ICU-acquired weakness was defined as an MRC sum-score less than 48, and severe weakness as a sum-score less than 36 [35–37]. Cough strength was assessed both at order and at suctioning and scored using a semi-quantitative 5-Likert scale as previously reported in other studies [13, 17]. It was graded 0 (absent), 1 (weak and ineffective), 2 (moderate), 3 (good and effective), or 4 (strong and very effective) and then defined as “ineffective” for a score of 0 to 1. Likewise, sputum amount was graded from 0 to 4 according to the following definition: 0 (absence), 1 (low quantity), 2 (moderate quantity), 3 (abundant), or 4 (very abundant) and then defined as “abundant” for a score of 3 to 4.

### Outcome

The primary outcome was extubation failure defined as reintubation or death within the first 7 days following extubation.

### Statistical analysis

Continuous variables were expressed as mean ± standard deviation (SD) or median [25th–75th percentiles], and qualitative variables were expressed as number and percentage. Continuous variables were compared using Student’s *t* test or the Mann-Whitney *U* test according to their distribution and categorical variables were compared using the chi-squared test or the Fischer’s exact test as appropriate.

Kaplan-Meier curves were plotted to assess time from extubation to reintubation or death within the 7 days following extubation and were compared by means of the log-rank test between patients with ineffective cough and the others and between patients with no ICU-acquired weakness and those with moderate or severe limb weakness.

Variables associated with extubation failure were assessed by means of multivariate logistic regression analyses. A backward manual selection procedure was performed for the maximal model using all factors associated with extubation failure with a *p* value < 0.15. Interactions were tested before performing the multivariate logistic regression and we verified that there was no multi-collinearity. The final model included variables significantly associated with extubation failure. We planned to analyze at least 50 extubation failure events, i.e., at least 300 extubations based on an extubation failure rate around 15–20%, in order to potentially enter at least the 5 following variables into the maximal model: severity at admission, duration of mechanical ventilation prior to extubation, ICU-acquired weakness, ineffective cough, and abundant secretions.

The correlation between MRC sum-score and cough strength was computed using the Spearman’s rank correlation test. A two-tailed *p* value of less than 0.05 was considered to indicate statistical significance. We used SAS software, version 9.4 (SAS Institute), and R software (available online at http://www.R-project.org) for all the analyses.

## Results

### Study population

Of the 416 patients included in the two prospective studies, 72 were excluded because they were at low-risk of extubation failure (*n* = 52) or had no assessment of cough strength or limb muscle strength (*n* = 20). As a result, 344 patients at high risk of reintubation were retained in the final analysis (see the figure showing a flow chart of the patients in the Additional file 1). Median duration of mechanical ventilation prior to extubation was 7 days (IQR 3–12]. All in all, 226 patients (66%) had underlying chronic cardiac or lung disease, 220 patients (64%) were older than 65 years, and 155 (45%) had been intubated for more than 7 days prior to extubation, including 47 patients (14%) of less than 65 years without any underlying cardiac or lung disease.

### Factors associated with extubation failure

The proportion of patients with extubation failure (reintubation or death at day 7) was 16% (56 out of 344 patients) (Table 1). Among them, 53 were reintubated and 3 patients died without reintubation within the 7 days following extubation. The main reason for reintubation was severe respiratory failure in 77% of patients (41/53). Patients who failed extubation were more severe, as indicated by higher SAPS II at admission, and had been intubated for a longer duration prior to extubation than those who succeeded extubation. They also had lower MRC sum-score and were more likely to have severe limb weakness, ineffective cough, and abundant secretions.

**Table 1:** Comparison of patients between success and failure of extubation defined as reintubation or death within the first 7 days following extubation

Variables	Extubation success (***N*** = 288)	Extubation failure (***N*** = 56)	***p*** value
**Characteristics of the patients at admission**			
Age, years	67 ± 14	70 ± 12	0.1068
Male sex, *n* (%)	172 (60%)	37 (66%)	0.3733
Body-mass index, kg/m^2^	28 ± 7	27 ± 5	0.4739
SAPS II at admission, points	47 ± 18	55 ± 20	**0.0042**
Underlying chronic cardiac disease, n (%)	153 (53%)	24 (43%)	0.1595
- Ischemic cardiopathy, *n*	83	15	
- Left ventricular dysfunction, *n*	66	12	
- Atrial fibrillation, *n*	58	11	
- History of cardiogenic pulmonary edema, *n*	29	4	
Underlying chronic lung disease, n (%)	84 (29%)	15 (27%)	0.7188
- Chronic obstructive pulmonary disease, *n*	58	13	
- Non-obstructive pulmonary disease, *n*	26	2	
Main reason for intubation			0.9180
- Acute respiratory failure, *n* (%)	130 (45%)	27 (48%)	
- Coma, *n* (%)	56 (19%)	8 (14%)	
- Shock, *n* (%)	36 (13%)	8 (14%)	
- Cardiac arrest, *n* (%)	12 (4%)	2 (4%)	
- Surgery, *n* (%)	54 (19%)	11 (20%)	
**Characteristics at time of extubation**			
SBT performed using T-piece, *n* (%)	148 (51%)	30 (54%)	0.7649
SBT performed using low levels of pressure-support, *n* (%)	140 (49%)	26 (48%)	0.7649
Median duration of MV prior to extubation, days	6 [3–12]	8 [5–15]	**0.0208**
Duration of MV > 7 days prior to extubation, *n* (%)	125 (43%)	30 (54%)	0.1617
MRC (Medical Research Council) score, points	49 ± 13	41 ± 16	**< 0.001**
Limb weakness (MRC sum-score < 48), *n* (%)	99 (34%)	31 (55%)	**0.0036**
- Moderate limb weakness (36 ≤ MRC sum-score < 48), *n* (%)	53 (18%)	12 (21%)	0.5966
- Severe limb weakness (MRC sum-score < 36), *n* (%)	46 (16%)	19 (34%)	**0.0022**
Ineffective cough, *n* (%)	16 (6%)	11 (20%)	**0.0008**
Abundant secretions, *n* (%)	87 (30%)	25 (45%)	**0.0301**
Patients with effective cough and without ICU-acquired weakness, *n* (%)	184 (64%)	24 (43%)	**< 0.001**
Prophylactic non-invasive ventilation after extubation, *n* (%)	173 (60%)	40 (71%)	0.1035
**Outcomes**			
- Median duration of mechanical ventilation, days	6 [3–12]	18 [13–28]	< 0.001
- Median length of ICU stay, days	11 [6–18]	24 [16–37]	< 0.001
- ICU mortality, *n* (%)	6 (2%)	26 (26%)	< 0.001

Values are given in mean ± standard deviation or median [interquartile range 25th–75th percentiles]

*SAPS* Simplified Acute Physiological Score, *SBT* spontaneous breathing trial, *MV* mechanical ventilation, *ICU* intensive care unit

*The same patient may have several underlying chronic cardiac diseases

**Fig. 1:**
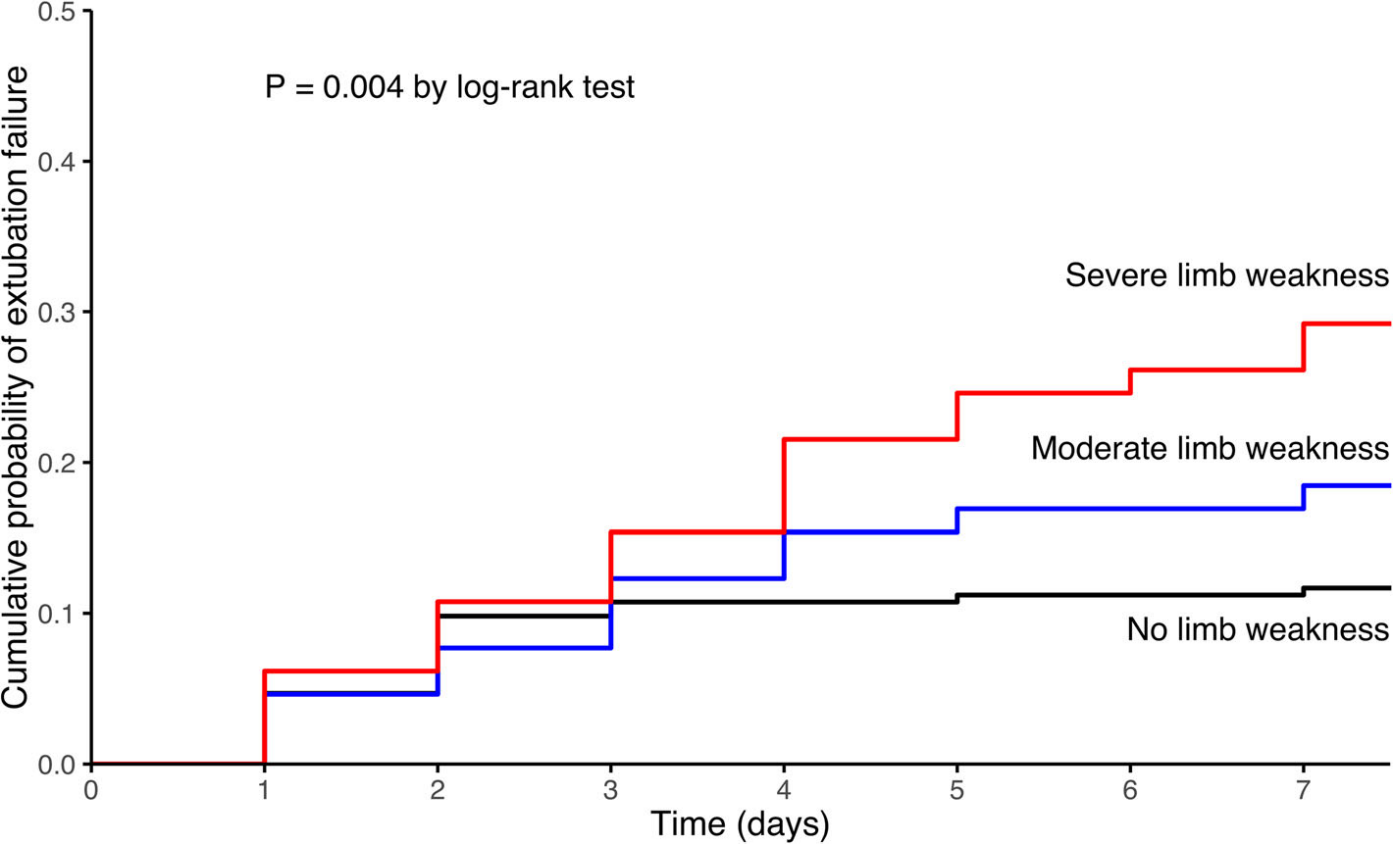
Kaplan-Meier curves of the cumulative probability of extubation failure defined as reintubation or death from extubation to day 7 in patients with no limb weakness (MRC sum-score ≥ 48 points) represented by the black line, moderate limb weakness (MRC sum-score ≥ 36 and below 48 points) represented by the blue line, and severe limb weakness (MRC sum-score < 36 points) represented by the red line

**Fig. 2:**
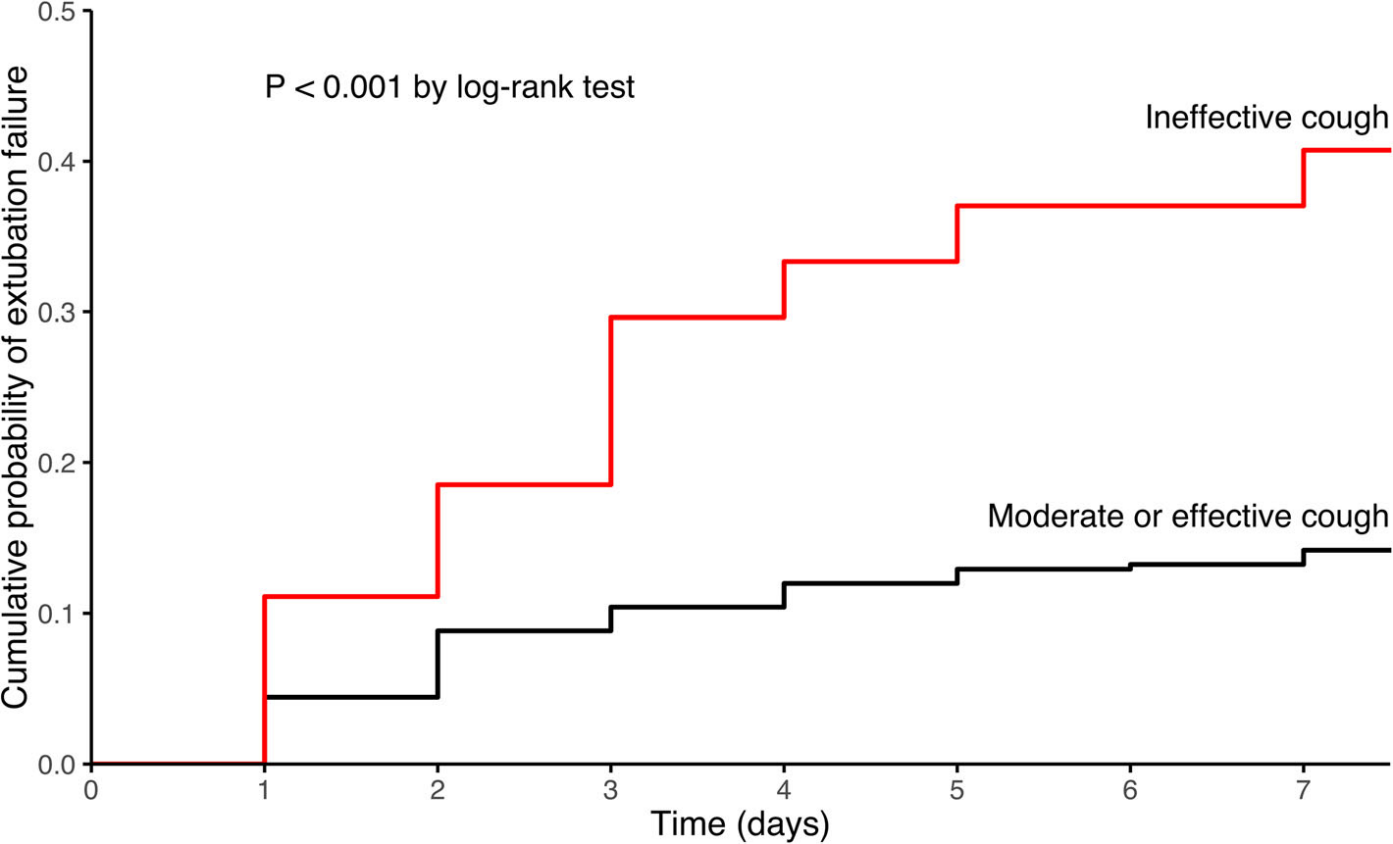
Kaplan-Meier curves of the cumulative probability of extubation failure defined as reintubation or death from extubation to day 7 in patients with ineffective cough (red line) and in those with moderate or effective cough (black line)

**Fig. 3:**
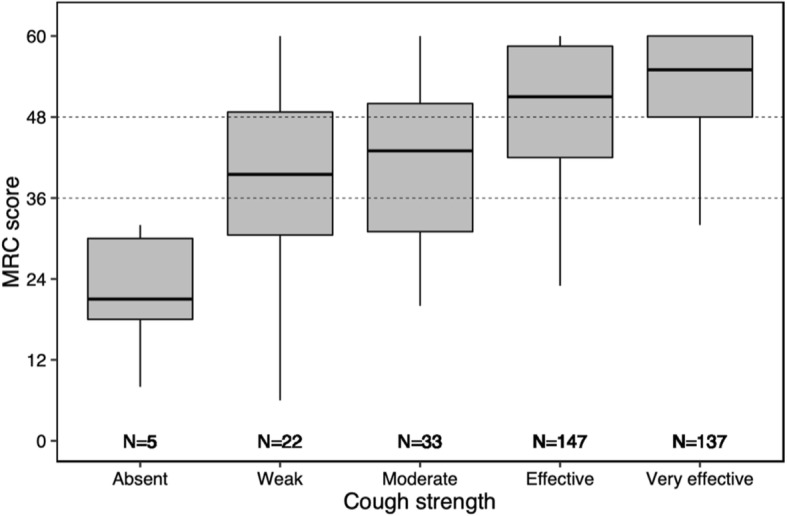
Box plots showing median MRC sum-score (25th–75th percentiles) according to cough strength considered as absent, weak or ineffective, moderate, effective, and very effective. MRC sum-score and cough strength were weakly but significantly correlated (rho 0.28; *p* < .001 using Spearman’s test)

ICU-acquired weakness was diagnosed in 130 out of the 344 patients (38%) and was classified as severe in half of them (65 out of 130 patients). Extubation failure rate was 12% (25 out of 214 patients) in patients with no limb muscle weakness compared with 18% (12 out of 65 patients) in those with moderate limb weakness and 29% (19 out of 65 patients) in those with severe limb weakness (*p* = 0.003 using chi-squared test and *p* = 0.004 using log-rank test) (Fig. 1). Cough was classified as ineffective in 27 patients (8%). The extubation failure rate was 41% (11 out of 27 patients) in patients with ineffective cough vs. 5% (16 out of 317 patients) in patients with effective or moderate cough strength (*p* < 0.001 using chi-squared test and log-rank test) (Fig. 2). MRC sum-score and cough strength were weakly but significantly correlated (rho = 0.28, *p* < 0.001) (Fig. 3). ICU-acquired weakness was diagnosed in 78% (21/27) among patients with ineffective cough as compared to 34% (109/317) in those with effective cough or moderate (*p* < 0.001). Cough was ineffective in 23% (15/65) among patients with severe limb weakness as compared to 9% (6/65) in those with moderate limb weakness and 3% (6/214) in those with no limb weakness (*p* = 0.001).

### Multivariate analysis

According to the multivariate logistic regression model, low MRC sum-score, ineffective cough, and high severity score at ICU admission were the three variables independently associated with extubation failure. The lower the MRC sum-score the greater the risk of reintubation. By replacing MRC sum-score (continuous variable) with limb weakness as a categorical variable in the model, severe but not moderate limb weakness and ineffective cough were the two factors independently associated with extubation failure after adjustment on severity score at ICU admission **(**Table 2**)**.

**Table 2:** Multivariate logistic regression analysis of factors associated with extubation failure defined as reintubation or death within the first 7 days following extubation

Variables	Adjusted odds ratio* [95% confidence interval]	***p*** value
**Model A entering MRC sum-score as continuous variable** (*R*^2^ = 0.079)		
SAPS II at admission—for each point increase	1.02 [1.01–1.04]	0.0233
Ineffective cough	2.72 [1.11–6.62]	0.0281
MRC sum-score—for each point lost	1.03 [1.01–1.05]	0.0067
**Model B entering MRC according to MRC sum-score < 48 (ICU-acquired weakness) or ≥ 48 (reference)** (*R*^2^ = 0.069)		
SAPS II at admission—for each point increase	1.02 [1.01–1.04]	0.0121
Ineffective cough	3.04 [1.27–7.29]	0.0127
ICU-acquired limb weakness (MRC sum-score < 48)	1.89 [1.02–3.48]	0.0426
**Model C entering MRC according to moderate, severe or no weakness (MRC sum-score ≥ 48 as reference)** (*R*^2^ = 0.071)		
SAPS II at admission—for each point increase	1.02 [1.01–1.04]	0.0164
Ineffective cough	2.90 [1.19–7.00]	0.0187
Moderate weakness (48 < MRC sum-score ≥ 36)	1.60 [0.74–3.47]	0.2342
Severe weakness (MRC sum-score < 36)	2.19 [1.06–4.54]	0.0351

All variables associated with extubation failure with a *p* value < 0.15 were included in the model (*R*^2^ = 0.104, 1) SAPS II at admission (age was not included in the maximal model given this variable is already included in the SAPS II calculation), (2) duration of mechanical ventilation prior to extubation, (3) moderate and severe ICU-acquired limb weakness, (4) ineffective cough (vs. effective or moderate), (5) abundant secretions, and (6) use of prophylactic non-invasive ventilation after extubation

*SAPS II* Simplified Acute Physiological Score II, *ICU* intensive care unit, *MRC* Medical Research Council

*Values of adjusted odds ratio are from the final model including only variables independently associated with extubation failure

## Discussion

In this post hoc analysis, pooling two prospective studies ICU-acquired weakness (prevalence 38%) was a strong predictor of extubation failure. The lower the MRC sum-score the greater the risk of extubation failure while severe limb weakness was independently associated with extubation failure, even after adjustment on cough strength and disease severity.

### Influence of ICU-acquired weakness on extubation failure

In addition to limbs, ICU-acquired weakness may affect respiratory muscles and lead to subsequent weaning difficulties and prolonged duration of mechanical ventilation [23, 24, 26–28]. However, the actual influence of ICU-acquired weakness on extubation outcome has been poorly studied. In a previous study, critical illness polyneuropathy was diagnosed using an electromyogram in 64 patients with sepsis [29]. The rate of reintubation was 41% among patients with polyneuropathy and 13% in the others (*p* < 0.05), but there was no clinical assessment of limb muscle strength. In another study including 377 critically ill patients, the authors found that the lower the MRC sum-score the higher the risk of reintubation within the 7 days after extubation [30]. However, MRC sum-score was measured only for the elbows and knees with an overall score ranging from 0 to 20 points, and limb weakness was considered for a score below 10 points. Unlike this study, we measured MRC sum-score according to the usual method with an overall sum-score ranging from 0 to 60 points. Limb weakness was considered according to the usual threshold in patients with a MRC sum-score below 48 points [21, 35–37], while severe limb weakness was considered in patients with a MRC sum-score below 36 points [36], these scores having shown a good interobserver agreement. Moreover, cough strength was not assessed in these previous studies and could have been a confounding factor making the results uncertain. Indeed, assessment of cough strength is essential to determine the respective role of ineffective cough and ICU-acquired weakness in extubation failure.

Coughing mechanics are mainly determined by the contraction of expiratory muscles, especially abdominal muscles, and effective closure of the glottis [18, 19, 38, 39]. Cough strength was significantly correlated with limb strength and consequently, ineffective cough was uncommon in patients with no limb weakness. Respiratory muscle weakness is frequently associated with limb weakness [23, 24, 27], and altered expiratory strength may explain ineffective cough in patients with limb weakness. However, the majority of patients with severe limb weakness had effective cough. ICU-acquired weakness may affect all inspiratory/expiratory muscles and even pharyngeal muscles, which could lead to respiratory failure due to altered overall respiratory muscle strength [23, 24, 27] or to swallowing disorders [25], even in patients with adequate cough. This study demonstrates that ICU-acquired weakness clinically assessed by limb strength using an easy and usual score at bedside was independently associated with extubation failure. However, only severe but not moderate limb weakness was actually associated with extubation failure, even after adjustment on cough strength.

Our study pooled two prospective studies in which both ICU-acquired weakness and ineffective cough were significantly associated with extubation failure using univariate analysis [15, 16]. However, after multivariate logistic regression, ineffective cough remained independently associated with extubation failure whereas this was not the case for limb weakness. This result was probably due to a lack of power in the abovementioned studies. Indeed, by pooling the two studies, cough strength and limb weakness both remained independently associated with extubation failure, even after adjustment on severity score.

### Strength and weakness of the study

Limitations of this study include its post hoc design and inherent bias and the absence of an objective measurement of cough strength. Cough strength was assessed by a physiotherapist using a semi-quantitative scale, and such subjective assessment of cough could limit external validity, especially for reproducibility of measurement and applicability in other centers. Moreover, as physiotherapists assessed both limb and cough strength, the prior diagnosis of limb weakness might have influenced subjective assessment of cough strength. However, several studies have reported an increased risk of extubation failure in patients with ineffective cough [6–17], whether cough is subjectively assessed using a semi-quantitative scale as our study [13–17] or objectively assessed by measuring cough peak flow using a spirometer [6–11, 16, 17], or measured using change in intra-abdominal pressure during tracheal aspirations [12]. In one of the previous studies, cough strength assessed using a semi-quantitative scale was significantly associated with extubation failure whereas cough peak flow was not, suggesting that the subjective assessment of cough strength might be more relevant than a single point measurement with a spirometer [16]. Cough peak flow is only one of the determinants of cough efficacy, and its measurement enables assessment only of voluntary cough strength which requires patients’ cooperation and may be a major limitation in ICU patients [6–11, 16, 17]. By contrast, semi-quantitative scales combine voluntary cough strength and involuntary cough strength during aspirations and could be assessed in all patients at the time of extubation [13–17]. Nevertheless, lastly, regardless of cough assessment method, physiological effective cough requires hermetic closure of the glottis which is not possible in an intubated patient.

Another limitation is that we did not assess respiratory muscle strength. Although the severity of limb weakness seems weakly correlated with diaphragm dysfunction, the majority of patients with ICU-acquired limb weakness (60 to 80%) have respiratory muscle weakness [23, 27]. The increased risk of extubation failure in patients with ICU-acquired weakness may be directly linked to respiratory muscle weakness. We previously found that diaphragm dysfunction assessed by ultrasound was not associated with an increased risk of extubation failure [16]. However, the overall respiratory muscle strength obviously depends not only on the diaphragm, but also on accessory respiratory muscles that could have a major role in patients at high risk of respiratory failure. That said, the assessment of limb weakness may be easy at bedside and seems to be a strong predictor of extubation failure, with no other measurement of respiratory muscle function. Last, measurement reliability of MRC sum-score has mainly been assessed in cooperative patients and could less accurate in patients still asleep or with delirium [40].

Strengths of the study include a prospective collection of data with a weaning protocol to assess readiness for extubation and systematic assessment of limb weakness by a physiotherapist before extubation. Moreover, the focus of the study on a homogeneous population of patients at high risk of reintubation may help to better generalize these results to other ICUs.

### Clinical implications

The study suggests that systematic assessment of ICU-acquired weakness using MRC sum-score should be daily integrated in the decision to extubate, in order to identify patients at high risk and to apply an appropriate strategy of ventilatory support after extubation. Prophylactic non-invasive ventilation immediately after extubation may be an optimal strategy of oxygenation to avoid reintubation in patients at high risk of extubation failure [4, 5]. Likewise, prophylactic non-invasive ventilation may be beneficial by avoiding reintubation in patients with ineffective cough, whereas it seems to have no beneficial effects in those with effective cough [10]. However, whether the application of non-invasive ventilation is efficient in patients with ICU-acquired weakness calls for further research.

## Conclusions

In our study, severe limb weakness and ineffective cough were both independently associated with extubation failure in patients at high risk of reintubation, even after adjustment on severity.

## Supplementary information

**Additional file 1.** Flow chart of the patients.

## Data Availability

Not applicable
