# Supplementary material for: Role of ICU-acquired weakness on extubation outcome among patients at high risk of reintubation
Source: Crit Care. 2020 Mar 12;24:86. doi: 10.1186/s13054-020-2807-9 (PMC7069045; doi:10.1186/s13054-020-2807-9)
Supplement: Supplementary file 1 — Additional file 1. Flow chart of the patients. [file 13054_2020_2807_MOESM1_ESM.pptx]

## Slide 1
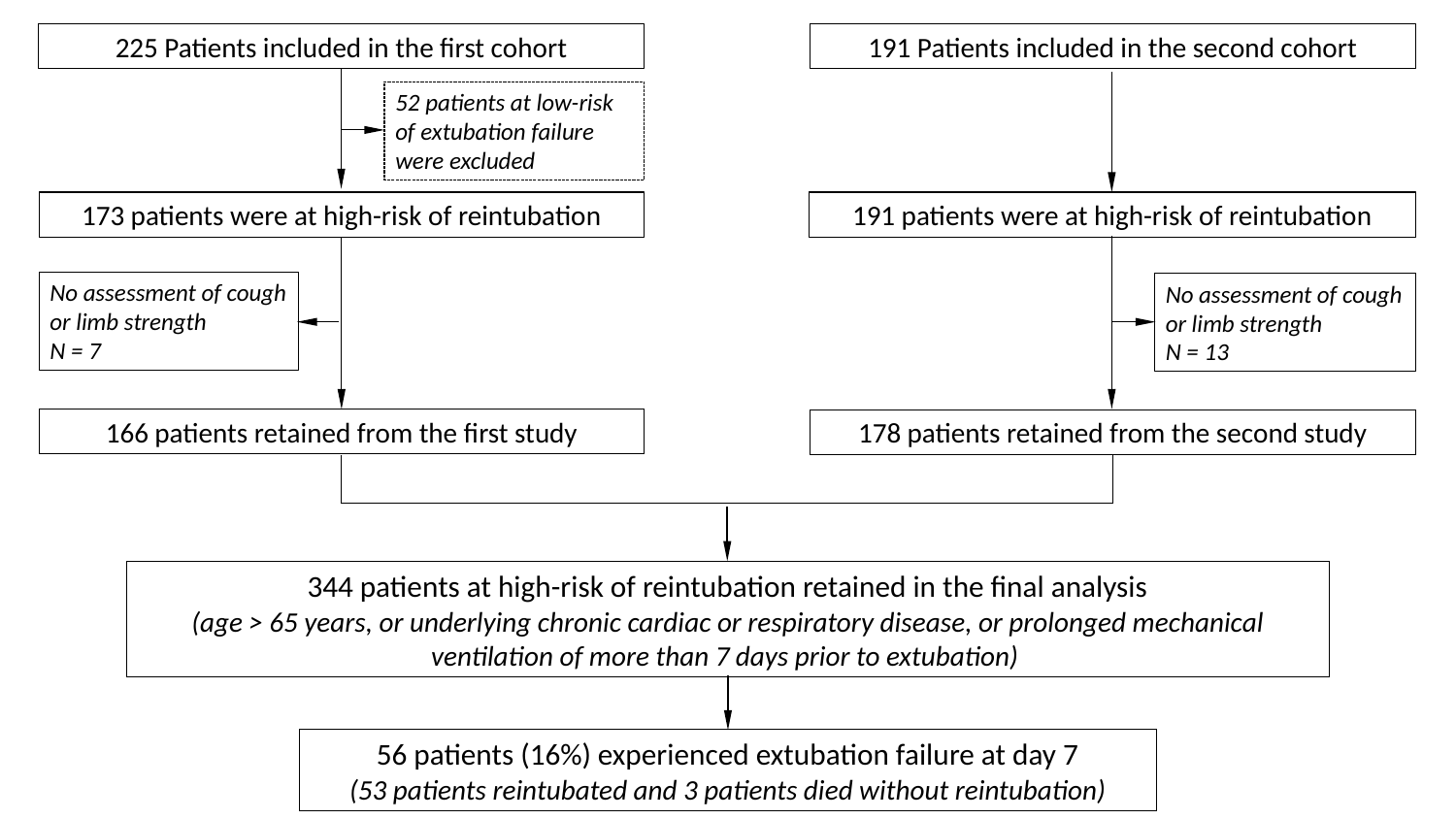

225 Patients included in the first cohort
191 Patients included in the second cohort
52 patients at low-risk of extubation failure were excluded
173 patients were at high-risk of reintubation
191 patients were at high-risk of reintubation
No assessment of cough or limb strength
N = 7
No assessment of cough or limb strength
N = 13
166 patients retained from the first study
178 patients retained from the second study
344 patients at high-risk of reintubation retained in the final analysis
(age > 65 years, or underlying chronic cardiac or respiratory disease, or prolonged mechanical ventilation of more than 7 days prior to extubation)
56 patients (16%) experienced extubation failure at day 7
(53 patients reintubated and 3 patients died without reintubation)
